# The morphology of the immature stages of *Squamapion atomarium* (Kirby, 1808) (Coleoptera, Brentidae) and notes on its life cycle

**DOI:** 10.3897/zookeys.892.36027

**Published:** 2019-11-27

**Authors:** Krzysztof Pawlęga, Jacek Łętowski, Ewelina Szwaj, Tomasz Gosławski

**Affiliations:** 1 Department of Zoology and Animal Ecology, University of Life Sciences in Lublin, Akademicka 13, 20–950 Lublin, Poland University of Life Sciences in Lublin Lublin Poland; 2 Braci Wieniawskich 6/59, 20–844 Lublin, Poland Unaffiliated Lublin Poland; 3 Puławskiego 8/51, 91–021 Łódź, Poland Unaffiliated Łódź Poland

**Keywords:** Apioninae, biology, central Europe, egg, host plant, life cycle, morphology, weevil

## Abstract

The immature stages (egg, mature larva and pupa) of *Squamapion atomarium* (Kirby, 1808), as well as its development cycle and the phenology of its developmental stages, are described for the first time. The larva and pupa of *S. atomarium* have typical morphological features of the subfamily Apioninae. Morphological data on the immature stages were compared with the only fully described *Squamapion* species, *S. elongatum* (Germar, 1817). The larvae of the two species differ in body size and shape, head shape, setae length, the chaetotaxy of the mouthparts, and individual types of setae on the pronotum and thorax. In the case of the pupa, there are also differences in body size and in the type of setae and chaetotaxy of the head, pronotum, metanotum and abdomen.

## Introduction

The genus *Squamapion* Bokor, 1923 belongs to the tribe Kalcapiini Alonso-Zarazaga, 1990 in the subfamily Apioninae Schönherr 1823 and family Brentidae Billberg 1820. The adult morphology, ecology, distribution and systematics of the Apionidae family have been presented in detail by Alonso-Zarazaga (1990, 2011), Petryszak (2004), Marvaldi and Lanteri (2005), Mokrzycki and Wanat (2005), Alonso-Zarazaga and Wanat (2014) and Alonso-Zarazaga et al. (2017). The immature stages of representatives of this family have been described by Scherf (1964), Łętowski (1991), Marvaldi (1999, 2003), Gosik et al. (2010), Wang et al. (2013), Oberprieler et al. (2014), and Łętowski et al. (2015). This genus is known from the Palearctic and Ethiopian regions and is poorly represented in the Oriental region. There are 33 known species in the Palearctic region, 19 in Europe, and only 9 in Poland (Mokrzycki and Wanat 2005; Alonso-Zarazaga 2011; Alonso-Zarazaga et al. 2017). These are herbivorous mono- or oligophagous species feeding on plants from the Lamiaceae family, with a preference for the genera *Salvia* L., *Thymus* L., *Thymbra* L., *Mentha* L., *Origanum* L., *Prunella* L. and *Saccocalyx* Coss. & Durieu. Their larvae bore tunnels inside the roots and stems, occasionally causing galls (Alonso-Zarazaga 1990). Adults have a small body size ranging from 1.10 to 2.70 mm. They are found mainly in dry and thermophilic environments – in non-forested areas and on the edges of forests and bushes (Burakowski et al. 1992).

The biology of only one species and the morphology of its immature stages are known – *Squamapion elongatum* (Germar, 1817) (Łętowski et al. 2015).

This study is a continuation of research on representatives of this genus found in Poland. The authors describe the morphology of the third larval instar and pupa as well as issues concerning the development and ecology of *Squamapion atomarium* (Kirby, 1808).

According to the literature, this species prefers warm, sandy areas and is usually found in xerothermic grasslands (Burakowski et al. 1992). Its host plants are Breckland thyme (*Thymus serpyllum* L.) and broad-leaved thyme (*T. pulegioides* L.). As regards its biology, *S. atomarium* feeds on the upper part of the stem of these plants, causing oval cecidia 2–4 mm long and 2 mm wide (Burakowski et al. 1992).

## Material and methods

### Insect collection

The research material comprised developmental stages (egg, larvae, and pupa) of *S. atomarium*, isolated in the laboratory from field-collected plants described in the literature as hosts. The choice of study sites was based on faunistic data on the occurrence of *S. atomarium* as well as our own observations of potential habitats in the Lublin region of Poland (Cmoluch 1963, 1971, 1987, 1992; Gosik and Łętowski 2003; Łętowski et al. 2003; Łętowski 2008). The sites were as follows: 1. Okale near Kazimierz Dolny (51°18'11.0"N, 21°53'58.6"E), 2. Bochotnica (51°20'38"N, 22°00'05"E), 3. Lublin-Górki Czechowskie (51°15'47"N, 22°32'03"E), 4. Lublin (51°13'11.50"N, 22°32'04.38"E), 5. Trześniów near Lublin (51°16'20"N, 22°37'04.10"E), 6. Kolonia Pliszczyn (51°17'38"N, 22°37'38"E), 7. the Stawska Góra Reserve near Chełm (51°22'23"N, 23°24'11"E) and 8. the Żmudź Reserve (51°00'35"N, 23°40'14"E). Immature stages of *S. atomarium* were found at sites 3, 5 and 8. In the remaining sites, despite the presence of host plants, no specimens were found. The material was collected from May to August 2016 and 2017. To obtain immature stages of the species, plants were collected at the sites every 2–3 days. This frequency made it possible to study the development cycle of the species in its natural conditions. Breeding was also conducted in the laboratory. *Squamapion atomarium* adults were collected individually, directly from the host plant and from its immediate surroundings.

### Breeding

Adult specimens were placed in plastic containers covered with mesh – separately for *T. serpyllum* L. and *T. pulegioides* L. Wet filter paper was placed on the bottom of the containers to maintain a suitable moisture level, together with thyme. The stems were searched for signs of oviposition and eggs about every three days. Then immature stages were grown in Petri dishes in a growth chamber, in the following conditions: daytime minimum 25 °C, daytime maximum 35 °C, minimum at night 15 °C, maximum at night 20 °C, humidity (60%), light duration – day 14 h, night 10 h. Immature stages were also grown in 125 ml plastic containers stored under room conditions (25 °C with a 14:10 photoperiod). Filter paper soaked in water was placed on the bottom of the container to maintain moisture, together with thyme stems with galls. The closed containers were monitored daily for mould. This method produced better results in terms of larvae survival than the use of the Petri dishes proposed by Scherf (1964). In order to track development and acquire larval stages, 5 stems were randomly selected, the galls were cut open, and developmental stages were isolated from them.

### Morphological descriptions

The immature stages obtained by the methods described above were preserved in 70% ethyl alcohol. Two methods were used to prepare microscope slides, as described by Łętowski (1991) and Gosik et al. (2010). To prepare the drawings, we used an OLYMPUS SZX12 and DP72 microscope at magnifications from 200× to 400× and a TESCAN VEGA3LMU scanning electron microscope (SEM) at magnifications from 500× to 2000×. The larvae for SEM images were subjected to critical point drying (CPD). Drawings based on the slides were made using Corel Draw 18.

The terminology of Marvaldi (1999, 2003) and Oberprieler et al. (2014) was used in the morphological descriptions of the larva and pupa for chaetotaxy, and the terminology of Zacharuk (1985) and Marvaldi (1998) for antennae. The number and distribution of setae are given for one side. Measurements of the head (following decapitation) were made on the head capsule, isolated from the body, with the mandibles closed. Measurements were made of 10 L_1_, 4 L_2_, 15 L_3_ and 10 pupae. The larvae were not separated by gender for the measurements. The mean and standard deviation for each parameter were calculated using Excel.

An analysis was made of the growth of the heads of individual larval instars based on Dyar’s law (1890), and the growth rate (GF) was determined based on Bednarz (1953).

### Morphological abbreviations

**AbI, AbVII, AbVIII, AbIX, AbX** – abdominal segments 1, 7–10, **ThI, ThII, ThIII** – thoracic segments 1–3, ***prns*** pronotal setae, ***pda*** pedal s., ***ps*** pedal s., ***eus*** eusternal s., ***lsts*** laterosternal s., ***prs*** prodorsal s., ***pds*** postdorsal s., ***as*** alar s., ***ss*** spicular s., ***eps*** epipleural s., ***ds*** dorsal s., ***les*** lateral epicranial s., ***fs*** frontal s., ***des*** dorsal epicranial s., ***pes*** posterior epicranial s., ***at*** antenna, ***Se*** sensorium, ***sb*** sensillum basiconicum, ***ss*** sensillum styloconicum, ***oc*** ocellus, ***enc*** endocarina. ***lrms*** labral setae, ***cls*** clypeus s., ***ams*** anteromedial s., ***als*** anterolateral s., ***mes*** median s., ***lr*** labral rods., ***mds*** – dorsal malae s., ***dms*** dorsal maxillary s., ***pfs*** palpiferal s., ***sts*** stipal s., ***mpxs*** maxillary palp s., ***mbs*** malar basiventral s., ***prms*** prelabium s., ***pms*** postlabium s., ***lgs*** ligular s., ***lbp*** labial palpus, ***as*** apical setae, ***ls*** lateral s., ***pls*** posterolateral s., ***sos*** suborbital s., ***rs*** rostral s., ***fes*** femoral s., ***ur*** urogomphi.

## Results

### Description of egg

Fig. 1

**Measurements** (in mm, *N* = 5). Length 0.28 (0.22–0.31), width 0.18 (0.15–0.20).

**Figure 1.**
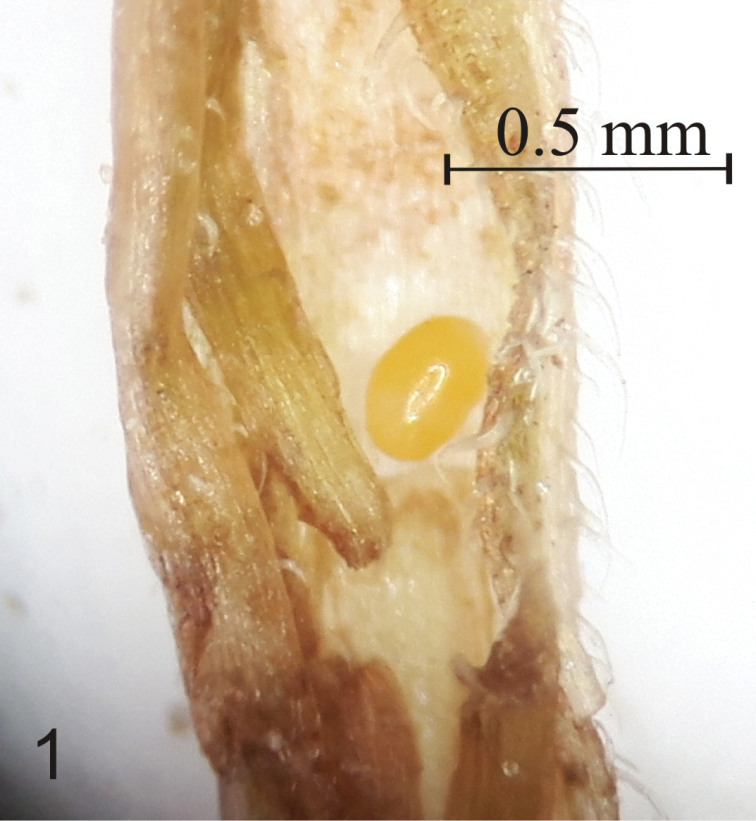
Egg of *Squamapion atomarium*.

**General.** Egg elliptical, shiny, smooth. Chorion soft, delicate (Fig. 1).

**Colouration.** Pale to dark yellow.

### Description of larva

Figs 2–6

**Measurements** (in mm). First larval instar (L_1_) – body length 0.46 (0.39–0.55), width 0.21 (0.19–0.23). Head width 0.13 (0.12–0.14).

Second larval instar (L_2_) – body length 0.68 (0.64–0.74). Body widest at abdominal segment III (0.34). Average pronotum width 0.27 (0.15–0.21). Head width 0.19 (0.17–0.21). Stemmata present.

Mature larva (third instar, L_3_) – body length 1.36 (1.09–1.72). Body widest at abdominal segment III (0.66, 0.50–0.86). Width of pronotum 0.47 (0.40–0.55). Head width 0.33 (0.30–0.38).

**Figure 2.**
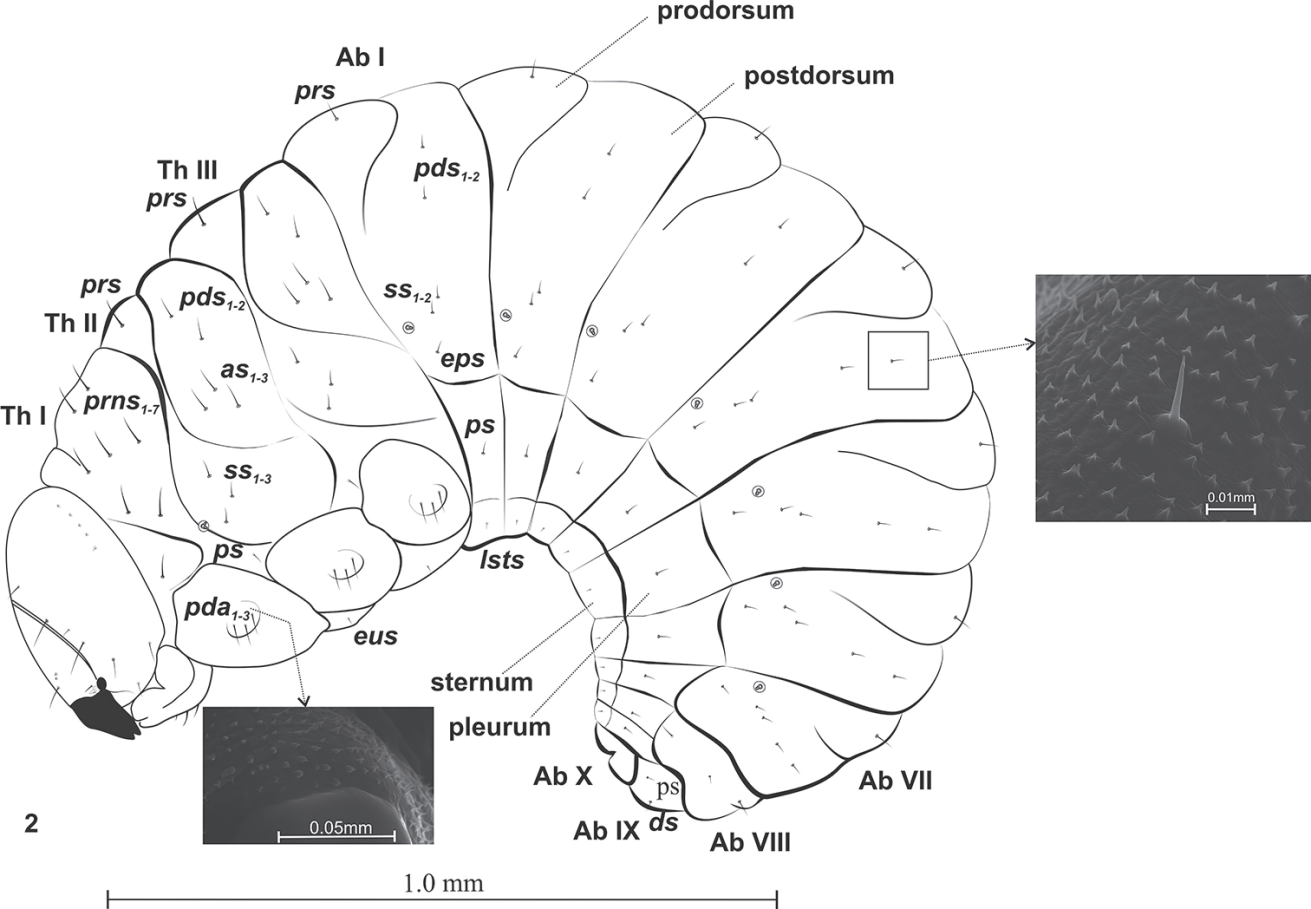
Mature larva (L_3_) of *Squamapion atomarium*, lateral view.

***General****L_3_*. Cylindrical, C-shaped, pale yellow with no distinct sclerotizations (Fig. 2). Cuticle microstructure of entire body with many small, sharply pointed cuticular structures. Thoracic and abdominal segments with characteristic, short setae. Body much narrower after abdominal segment VIII.

**Figure 3.**
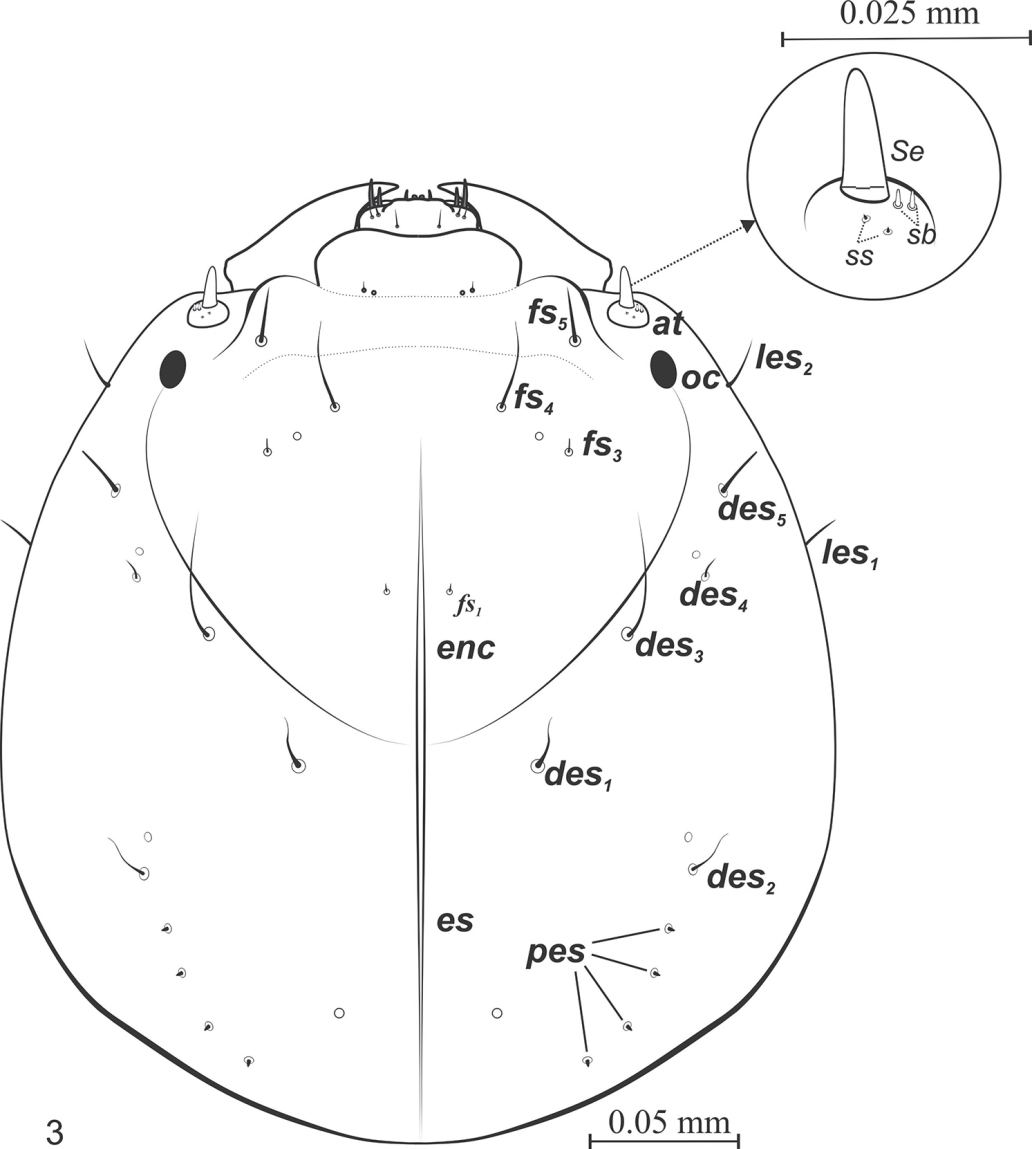
*Squamapion atomarium* (L_3_), – epicranium, dorsal view.

***Head*** (Fig. 3). Pale yellow, later dark yellow, slightly hidden in prothorax, longer than wide, slightly egg-shaped, widest at 2/5 of length. Epicranial suture visible. Endocarina (*enc*) distinct, long, together with epicranial suture extends 3/4 length of head (Fig. 3). End of frontal suture with distinct stemmata. Antennae (*at*) without articulations. Sensorium (Se) long, slightly narrowing apically. Antenna with 4 sensillae: 2 *sb* (*sensillum basiconicum*) and 2 *ss* (*sensillum styloconicum*) (Fig. 3). Epicranium with 2 lateral epicranial setae (*les1,2*). *Les1* more than 4 times longer than *les2*. Dorsal part of epicranium with 5 visible dorsal setae (*des1–5*), *des1,3–5* situated more or less along frontal suture, *des2* on extension of line of *pes* (Fig. 3). *Des3* very long, *des4* very short and *des1,2,5* short and more or less equidistant. Epicranium with 4 thorn-shaped posterior epicranial setae posterolaterally (*pes1–4*) –very short and more or less equidistant. Frons with 4 frontal setae (*fs1,3–5*) (Fig. 3). *Fs1,3* short (shortest of all *fs*), *fs1* situated by endocarina at about 1/3 length of frons, *fs3* situated outermost of all *fs*, close to *fs4*. Setae *fs4* long – longest of all *fs.* Setae *fs5* slightly above anterior margin of stemmata.

**Figure 4.**
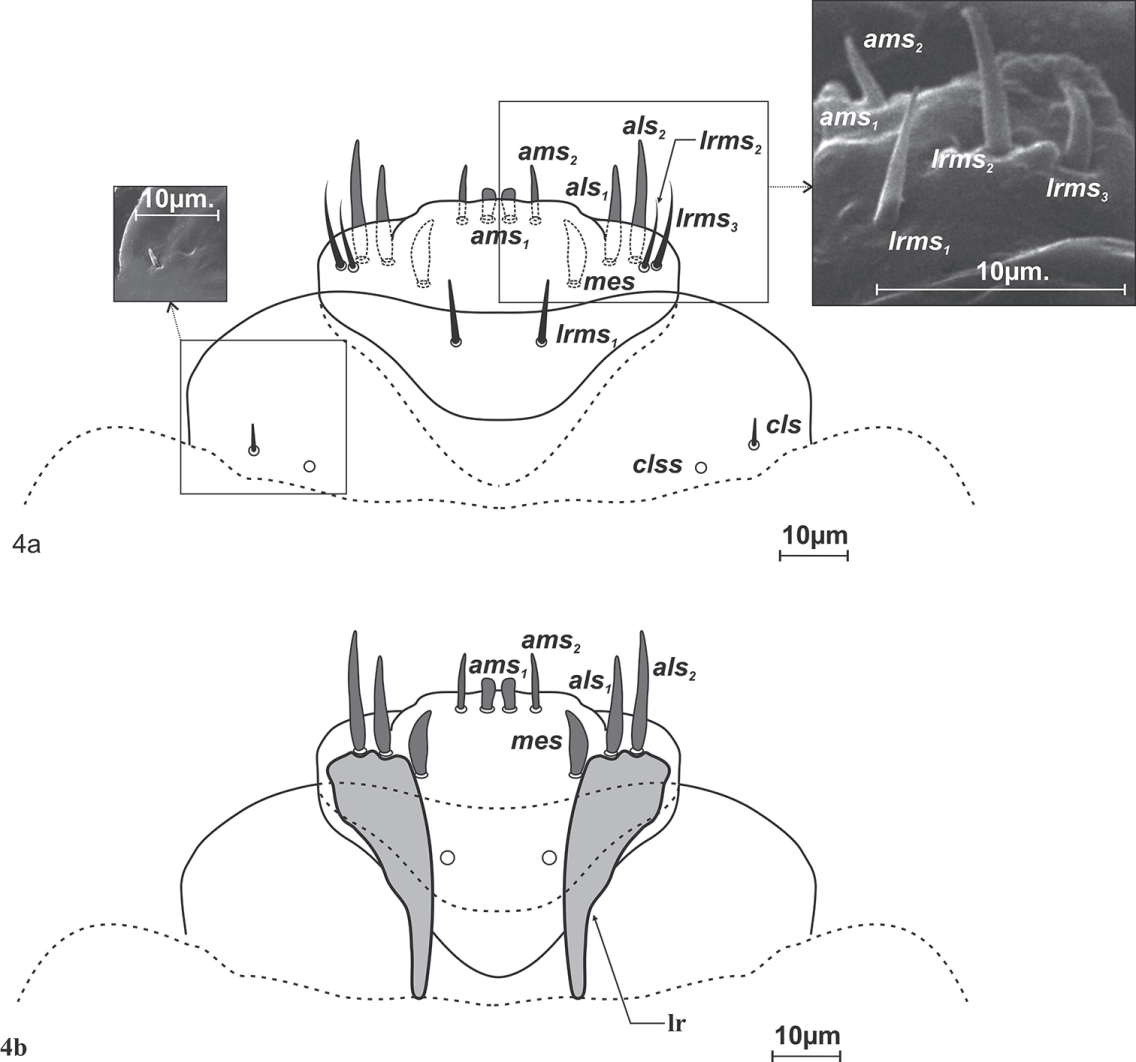
*Squamapion atomarium* (L_3_) labrum and clypeus: **a** dorsal view **b** ventral view.

**Figure 5.**
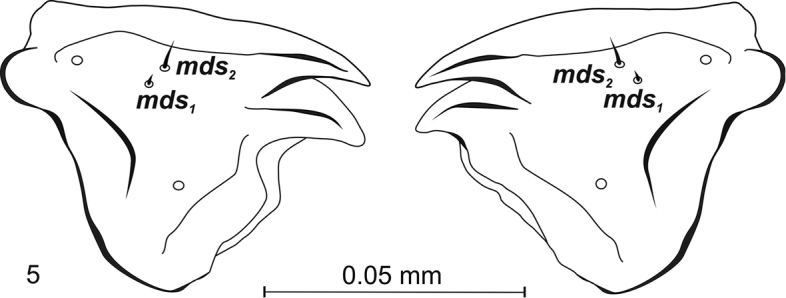
*Squamapion atomarium* (L_3_) – mandibulae.

**Figure 6.**
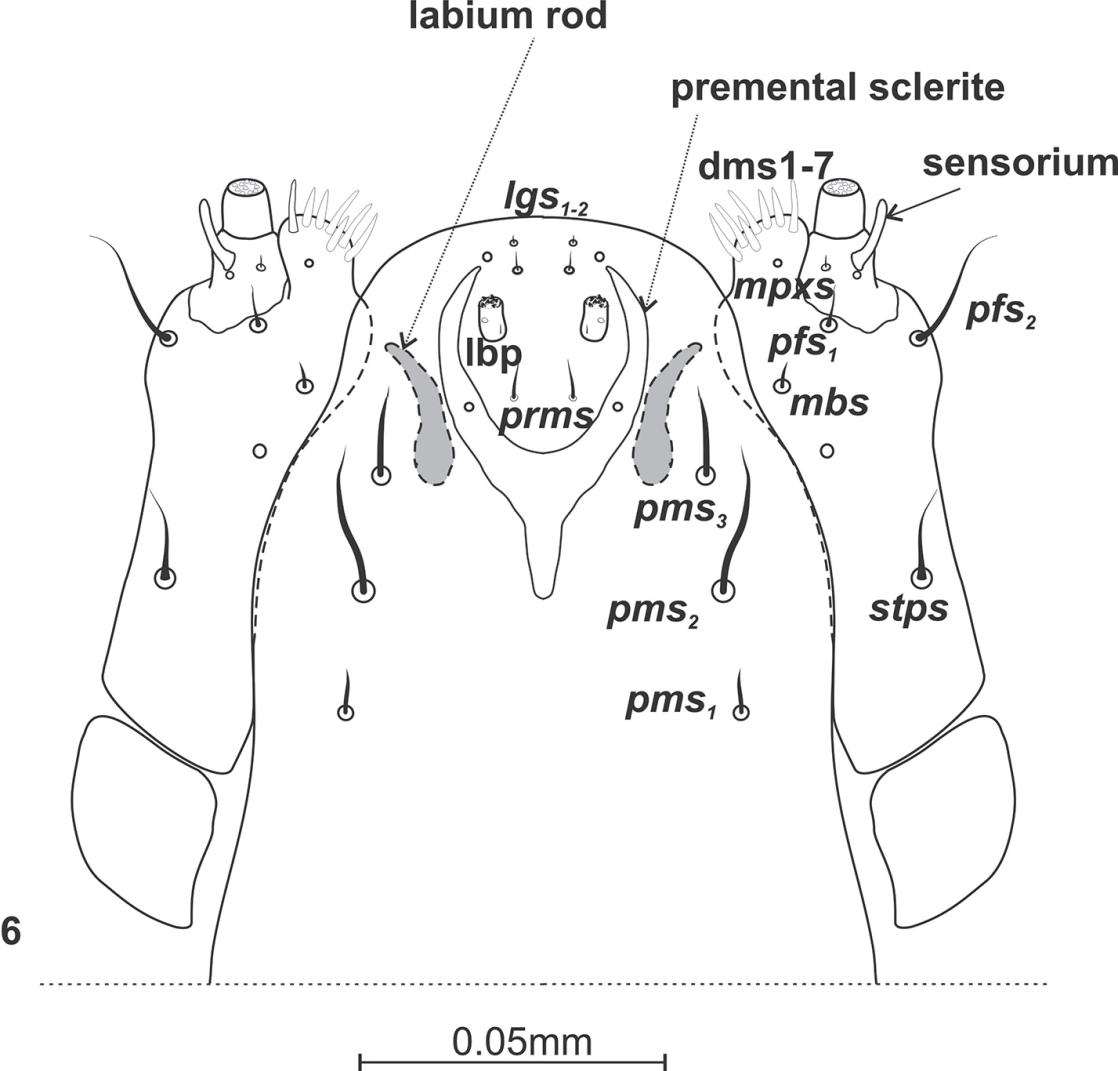
Labium and maxillae (L_3_) of *Squamapion atomarium*.

***Mouthparts*.** Labrum – anterior margin slightly arched. Dorsal side with 3 thorn-shaped labral setae (*lrms*), of which *lrms2* long and longer than others; *lrms_1_* closer to centre, below mid-height of labrum, *lrms2* and *lrms3* anterolaterally (Fig. 4a). Epipharynx anteriorly with 2 anteromedial setae (*ams*), of which medial *ams1* finger-shaped, outer setae *ams2* thorn-shaped (Fig. 4b). Beside *ams*, 2 *als* on epipharynx, arranged more or less diagonally from corner to centre of labrum. *Als1* slightly shorter than *als2*. Both wider at base and narrowing apically. *Mes* digitiform and placed antero-medially. Labral rods (*lr*) present, long, extending well beyond suture (Fig. 4a). Clypeus kidney-shaped, with slightly concave anterior margin. 1 short seta *cls* at lower margin, between them 1 sensillum (*clss*) (Fig. 4a). Mandible massive, highly sclerotization, light to dark brown in colour. Two teeth equal in size, curved. Dorsally 1 pair mandible dorsal setae (*mds1,2*) and 1 sensillum (Fig. 5). Setae close together, one above the other, each sensilla peripherally. *Mds2* more than twice longer than *mds1*. Inner margin of inner teeth serrated. Maxillary stipes elongated, widening apically, narrowed at mid-length, with 4 distinct, hair-like setae (Fig. 6). In lower part 1 stipal seta (*stps*). In the upper part palpiferal setae *pfs1* fairly short, placed centrally under maxillary palpus, *pfs2* very long, placed on inner side, basioventral seta (*mbs*) short. Maxillary palpus (*mp*) 2–segmented, distal segment cylindrical, smaller than basal segment, with 10 nodular cuticular tubercles situated apically. Basal segment with rod-shaped sensorium, 1 minute maxillary palp seta (*mpxs*) and 1 pore. Malar part of maxilla with 7 dorsal maxillary setae (*dms1–4, vms1–3*) clearly visible, finger-shaped setae of equal length in comb-like arrangement.

Labium cup-shaped (Fig. 6). Base of prementum rounded. Postmentum with 3 pairs postmental setae (*pms1–3*), distributed evenly, one over the other, closer to outer part of postmentum, more or less parallel to its edges. First pair setae (*pms1*) situated closest to lower margin, shortest of all *pms.* Above it *pms2*, very long and longest of *pms*, thick, narrowing only at apex. Setae *pms3* situated at 2/3 height of labium, similar in structure to *pms2* but half their length. Labium with Y-shaped premental sclerite situated centrally. 1 pair sensilla at base of arms of this structure. At height of premental sclerite, dorsally, chitinized inverted comma-shaped labial rods with uneven edges. Labium with 1 pair simple palpi (*lbp*), with 7 palpillae apically, 1 inner seta at base and 1 outer sensillum. In front of palpi 1 pair long premental setae (*prms*). Behind palpi 2 pairs very short ligular setae (*lgs*) and 1 pair sensilla (Fig. 6).

***Thorax*.** Thoracic segments with well visible pedal area. Thoracic setae longer than others. Prothorax with 7 pronotal setae varying in length (*prns*), 1 pleural seta (*ps*) and on pedal area 3 pedal setae (*pda*) (Fig. 2). Meso- and metathorax each with 1 relatively long prodorsal seta (*prs*), 2 postdorsal setae (*pds*), 3 alar setae (*as*), 3 spicular setae (*ss*), 3 *pda* on pedal area and 1 short eusternal seta (*eus*). Thoracic spiracle bicameral, located intersegmentally, between Th.I and Th.II (Fig. 2).

***Abdomen*.** Tergites I–VII with 2 folds and 1 seta (*prs*) on prodorsum. Postdorsum with 2 *pds* and 2 *ss* of varying size; 1 epipleural seta (*eps*) slightly below *ss.* Pleurum with 1 *ps.* Sternum with 1 laterosternal seta (*lsts*). Tergit VIII with gentle folds and with 1 *prs*, 1 dorsal seta (*ds*) and 1 *eps.* Tergit IX without folds, with 1 *ds* and 1 *ps.* Sternum and pleurum of segments VIII–IX with 1 *ps* and 1 *lsts*. Segments I–VII with unicameral spiracles, others without spiracles (Fig. 2).

### Description of pupa

Figs 7–10

**Figures 7–9.**
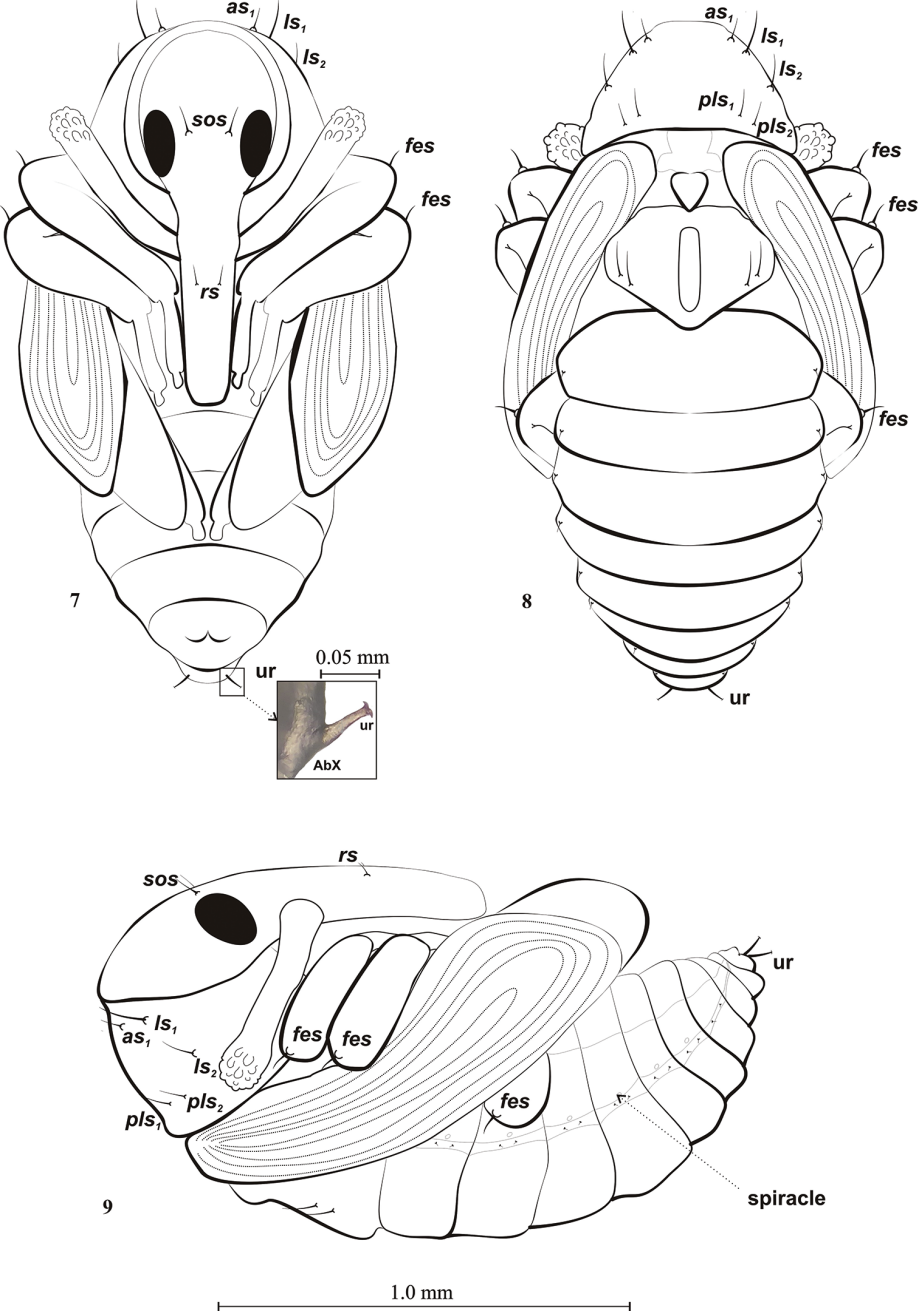
Pupa of *Squamapion atomarium***7** ventral view **8** dorsal view **9** lateral view.

**Figures 10–15.**
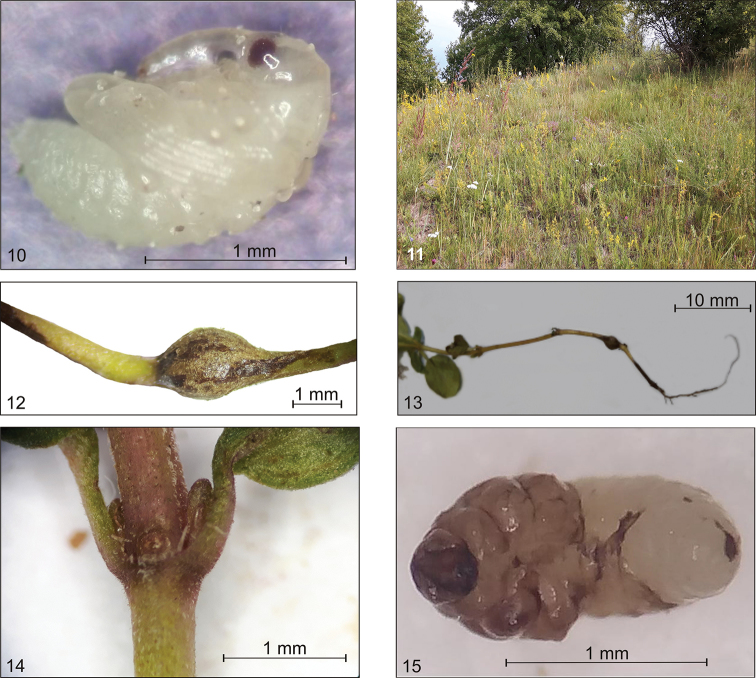
*Squamapion atomarium***10** pupa **11** occurrence environment **12** a gall **13** the most common place to lay eggs at the root collar **14** place for laying eggs **15** larva in prepupal stage for pupation.

**Measurements** (in mm). Body length 1.51 (1.24–1.63), width 0.84 (0.72–0.93) (Figs 7–10).

**Colouration.** Colour creamy-white.

***Head*.** Eyes large, with 1 supraorbital seta (*sos*) between them. Rostrum long, extending to end of tarsi of mesolegs, not very wide, with 1 rostral seta (*rs*) below base of antennae, shorter than *sos.* Antennae relatively long, club with conical papillae. Antennae sub-parallel to protibia (Figs 7, 9).

***Thorax*.** Pronotum wider than long; sides with 2 lateral setae – long *ls1* and shorter *ls2*; 1 apical seta on apex (*as*), half length of *ls1* (Figs 7, 8); lower margin with 2 posterolateral setae (*pls1,2*), similar in length to *as1*. Mesonotum without setae. Metanotum with 2 setae, slightly shorter than *ls1* (Figs 8, 9). Each femur with 1 femoral seta (*fes*) on convex base (Figs 8, 9).

***Abdomen*.** Chaetotaxy very sparse. Each segment with 1 short dorsal seta located close to lateral margin. Each of lateral parts of abdominal segments I–VII with 1 pair minute lateral setae. Spiracles located between tergites and pleurites, clearly visible on segments I–VI, on others absent (Fig. 9). Segment IX terminally with 1 pair urogomphi (ur) with characteristic ends in form of flattened bifurcation (Fig. 7).

### Notes on biology and life cycle

Figs 11–17

**Host plant.** The life cycle of *S. atomarium* was described based on field data and laboratory observations. *Thymus serpyllum* and *T. pulegioides* were confirmed as host plants (Fig. 11).

**Figure 16.**
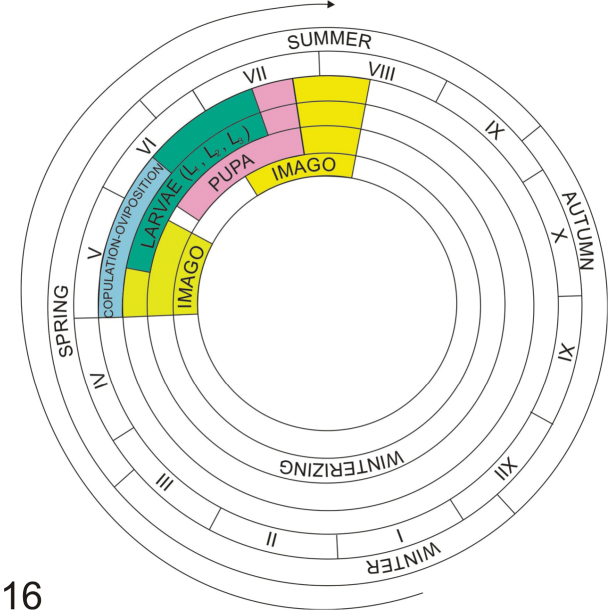
Life cycle of *Squamapion atomarium*.

**Life cycle.** Adults, following overwintering and maturation feeding, begin copulation and egg laying in the first half of May. Increased egg laying was observed at the end of May, and single eggs were still noted in early June. Adults usually feed in the evening, by gnawing round holes in the leaf that do not exceed 1 mm in diameter. The fertilized female gnaws a cavity in the stem and lays one egg in it (Fig. 12). Oviposition takes place primarily at the root collar, but it was also observed up to the fourth or fifth node, in both nodes and internodes (Fig. 13). After laying the egg the female does not seal the site with any secretion. The first instar larva (L_1_) hatches on average 4 days after the egg is laid and moults after 10–12 days. The L_1_ instar was observed as early as mid-May, but these were isolated specimens. Maximum emergence was observed from the second third of May. L_1_ larvae were found until mid-June. The second larval instar (L_2_) appeared at the end of May. The activity of L_2_ larvae causes distinct galls about 1.32 mm long and about 0.75 mm wide. Furthermore, L_2_ gnaws out an opening for oviposition on the opposite side of the groove, but does not gnaw through the skin. The second larval instar lasts on average 10 days, and then the larva moults again. L_3_ larvae appeared as early as the last third of June and were noted until mid-July. The average duration of this stage is about 11 days. This stage continues feeding and the gall grows, reaching on average about ca 2.31 in length and ca 1.70 mm in width (Fig. 14). The third larval instar enlarges the opening in the stem. Then pupation takes place (Fig. 15). The pupal stage lasts 2–3 days on average. The first pupae appeared at the end of June. Finally, at a maximum 40 days after the egg is laid, adult individuals appear. An increase in the emergence of adults took place from mid-July. The entire life cycle of *S. atomarium* is presented in the diagram in Figure 16.

**Figure 17.**
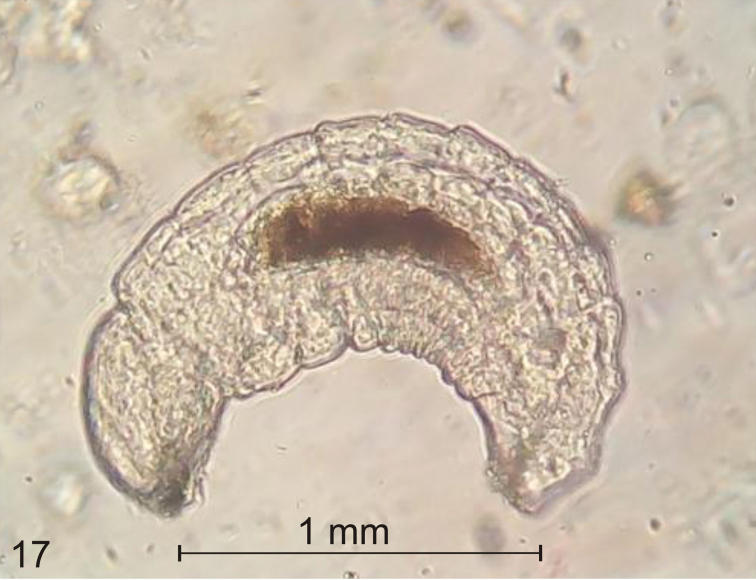
Larva of a chalcidoid endoparasitoid found inside the mature larva (L_3_) of *Squamapion atomarium*.

**Parasitoids.** In the second half of July, endoparasitic hymenopterans of the superfamily Chalcidoidea were very active, which is manifested by the high level of parasitism of L_3_ larvae. On average 7 of 10 third-instar larvae exhibited symptoms of parasite infection: dark red discolouration on the thoracic tergites and pleurites and swelling of the abdominal segments caused by the growth of the intruder larvae (Fig. 17). The mature larva of the parasitoid usually occupied the space from the second or third thoracic segment to the eighth abdominal segment. The adult larva of the parasite is ca 0.75 mm long and ca 0.56 mm wide. The body of the pupa of the parasitoid is black with a metallic sheen and well chitinized. The parasites brought about the death of L_3_ of *S. atomarium*.

### Head growth of larval instars and growth factor (GF)

Figs 18, 19

**Figure 18.**
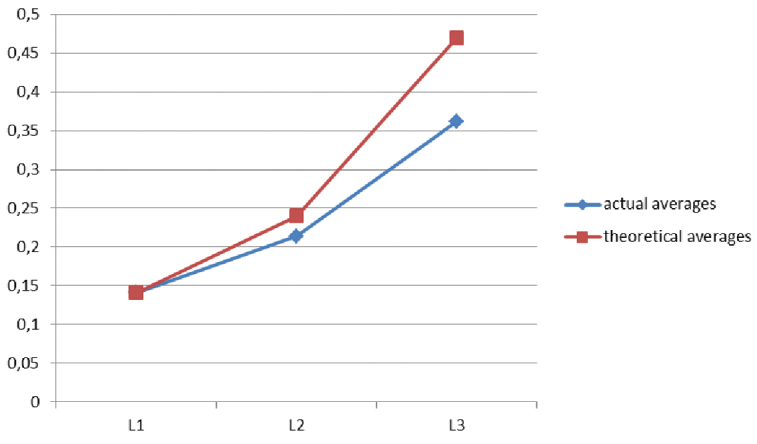
Mean real and average theoretical head lengths of *Squamapion atomarium* larval stages.

**Figure 19.**
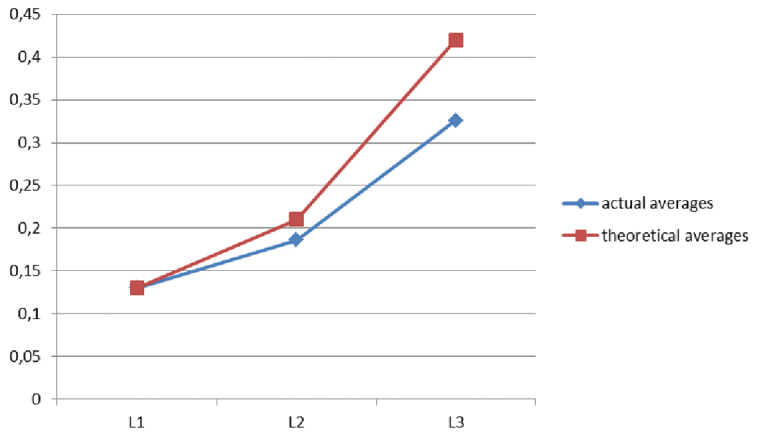
Mean real and average theoretical head widths of *Squamapion atomarium* larval stages.

Deviations of the mean dimensions of the heads of individual larval stages from the theoretical dimensions are shown in Figures 18, 19. Analysis of the ratios of the head sizes of larval instars does not clearly result in a single growth factor. GF between L_1_ and L_2_ is 1.43 and between L_2_ and L_3_ it is 1.75.

## Discussion

Among species of the genus *Squamapion*, only *S. elongatum* (Germar, 1817) has previously been described, and the existing data on *S. atomarium* concern only its habitat and host plants, with an equal role ascribed to *T. serpyllum* and *T. pulegioides* (Burakowski et al. 1992; Łętowski et al. 2015). The present study has shown that the preferred plant species is broad-leaved thyme (*T. pulegioides*), on which more galls were observed. This is most likely linked to the environment inhabited by *S. atomarium*, where this species of thyme is more common. Another new observation is the site of oviposition and galls. According to Burakowski et al. (1992), the larva feeds on the upper part of the stem. In the present study, the eggs were usually laid in the lower part of the stem.

The morphology of the L_3_ larva and pupa of *S. atomarium* does not differ from the typical characters of the subfamily Apioninae (Alonso-Zarazaga and Wanat 2014). These features are the strongly convex and C-shaped body, colour, subglobose head, coronal suture and endocarinal line, clearly visible stemmata close to the frontal suture; numbers of *des, les* and *fs*; transverse and trapeziform clypeus with one pair of *cls* and one pair of *clss*; chaetotaxy of the labrum and epipharynx; mandible chaetotaxy; morphology and chaetotaxy of the maxilla and labium; thoracic segments with a prodorsum and postdorsum; very small prodorsum of the pronotum; morphology and chaetotaxy of the pro-, meso- and metanotum, except the number of *as*, with three pairs in *S. atomarium*; mesothoracic spiracles on the membrane between the pro- and mesothorax; and the abdominal morphology and chaetotaxy, except for the presence of *lsts* on the 8^th^ abdominal segment in *S. atomarium*.

Thus the immature stages of the species are generally very similar in their morphology to those described by Łętowski (1991) for *Synapion ebeninum* (Kirby, 1808), *Stenopterapion intermedium* (Eppelsheim, 1875) and *Metatrichapion reflexum* (Gyllenhal, 1833), by Gosik et al. (2010) for *Diplapion confluens* (Kirby, 1808), or by Wang et al. (2013) for *Pseudaspidapion botanicum* Alonso-Zarazaga & Wang, 2011. *Squamapion atomarium* was also confirmed to possess an apomorphic trait of Apioninae emphasized by Marvaldi (2003), namely a lack of spiracles beginning in the eighth abdominal segment. There are differences in body size and in the number and distribution of setae (see Łętowski et al. 2015).

In the comparative analysis of the egg and L_3_ larva of *S. atomarium* and *S. elongatum*, the two species are distinguished by differences in the size of both the egg and the L_3_ larvae – in *S. atomarium* they are about half the size as in *S. elongatum* (Łętowski et al. 2015). Similar differences are found in the width of the epicranium of the two species, the shape of the head, and some features of their chaetotaxy. The differences are presented in Table 1.

**Table 1.** Character comparison between L_3_*Squamapion atomarium* and *Squamapion elongatum*.

**Trait**		**Species**	
		***Squamapion atomarium***	***Squamapion elongatum***
Body mm (length/width)		ca 1.36/ca 0.66	ca 2.78/ca 1.24
Setae		shorter, with pointed ends	longer
Head		slightly egg-shaped	oval
Antennae		4 sensilla	2 sensilla
Number of setae on maxillary palpus	basal segment	1 seta, 1 sensillum	1 seta, 2 sensilla
	distal segment	none	1 short sensillum
Labrum/epipharynx	*ams*	2 pairs (*ams1–2*)	3 pairs(*ams1–3*)
	*als*	2 pairs	3 pairs
	*lr*	large, widening towards outer margin of epipharynx	narrow
Labium with *pms*		3 pairs(*pms1–3*)	2 pairs(*pms1* and *pms3*)
Number of conical papillae *dms*		4	5
Number of setae *prns* on pronotum		7	5
Number of setae *pda*		3	2
Number of setae *ss*		2	3

The case of the pupa is similar. There are clearly visible differences between species in body size and chaetotaxy. The body of the pupa of *S. atomarium* is shorter than that of *S. elongatum* (1.5–2.0 times) and has far fewer abdominal setae (Table 2). There are also minor differences in body colour. Similar proportions of body length are found in adults.

**Table 2.** Character comparison between the pupa of *Squamapion atomarium* and *Squamapion elongatum*.

**Trait**		**Species**	
		***Squamapion atomarium***	***Squamapion elongatum***
Body mm (length/width)		ca 1.51/ca 0.84	ca 2.67/ca 0.94
Colour		creamy-white	whitish-grey
Head setae		1 pair *sos*, 1 pair *rs*	1 pair *vs*, 1 seta *rs*
Pronotum		1 pair *as* (*as1*), 2 pairs *ls* (*ls1,2*)	2 pairs *as* (*as1,2*), 1 pair *ls*
Metanotum		2 pairs of setae	3 pairs of setae
Abdomen	lateral part	only 1 pair of setae of I–VII abdominal segments	absent
	dorsal part	absent	AbI-III: 7 pairs,
			AbIV-VI: 5 pairs,
			AbVII: 3 pairs,
			AbVIII: 1 pair
Urogomphi		flattened bifurcation, straight	crescent-shaped, narrow

The study and descriptions of additional species of the genus *Squamapion* will make it possible to distinguish and describe its generic characters.

Analysis of the growth rate and the ratio of actual and theoretical average head sizes produced some discrepancies that may have been influenced by the fact that the individuals were not divided by sex or collection site, and thus may have represented different populations.

## References

[B1] Alonso-Zarazaga MA (1990) Revision of the supraspecific taxa in the Palaearctic Apionidae Schoenherr, 1823 (Coleoptera, Curculionoidea) (2. Subfamily Apioninae Schoenherr, 1823: Introduction, keys and descriptions. Graellsia 46: 19–156.

[B2] Alonso-Zarazaga MA (2011) Apionidae. In: Löbl I, Smetana A (Eds) Catalogue of Palaearctic Coleoptera (Vol. 7). Stenstrup, Apollo Books, 373 pp.

[B3] Alonso-Zarazaga MA Wanat M (2014) Apioninae Schoenherr, 1823. In: Leschen RAB Beutel RG (Eds) Handbook of Zoology/ Handbuch der Zoologie, Band 4: Arthropoda, 2.Hälfte: Insecta, Coleoptera, Beetles (Vol. 3). Morphology and Systematics (Phytophaga). De Gruyter, Berlin, Boston, 395–415.

[B4] Alonso-Zarazaga MA Barrios H Borovec R Bouchard P Caldara R Colonnelli E Gültekin L Hlavá P Korotyaev B Lyal CHC Machado A Meregalli M Pierotti H Ren L Sánchez-Ruiz M Sforzi A Silfverberg H Skuhrovec J Trýzna M Velázquez de Castro AJ Yunakov NN (2017) Cooperative catalogue of Palaearctic ColeopteraCurculionoidea. Monografías electrónicas S.E.A. (Vol. 8), 729 pp.

[B5] Bednarz S (1953) Wzrost głowy larw Tettigonia viridissima (Saltatoria, Tettiginiidae) a hipoteza Dyara. Polskie Pismo Entomologiczne, Warszawa, T. XXIII, 14: 191–203.

[B6] Burakowski B Mroczkowski M Stefańska J (1992) Katalog Fauny Polski. Cz. XXIII, T. 18. Chrząszcze – Coleoptera. Ryjkowcowate prócz ryjkowców – Curculionioidea prócz Curculionidae. Wydawnictwo Muzeum i Instytut Zoologii PAN, Warszawa, 324 pp.

[B7] Cmoluch Z (1963) Badania nad fauną ryjkowców (Coleoptera, Curculionidae) roślinnych zespołów kserotermicznych południowo-wschodniej części Wyżyny Lubelskiej. Annales Universitatis Mariae Curie-Skłodowska, sectio C 17(1): 1–75.

[B8] Cmoluch Z (1971) Studien über Rüsselkäfer (Coleoptera, Curculionidae) xerothermer Pflanzenassoziationen der Lubliner Hochebene. Acta Zoologica Cracoviensia 16: 29–216.

[B9] Cmoluch Z (1987) Ryjkowce (Coleoptera, Curculionidae) roślinnych zbiorowisk kserotermicznych Białej Góry koło Tomaszowa Lubelskiego. Annales Universitatis Mariae Curie-Skłodowska, sectio C 39: 187–197.

[B10] Cmoluch Z (1992) Rüsselkäfer (Coleoptera, Curculionidae) von Polesie Lubelskie. Annales Universitatis Mariae Curie-Skłodowska, sectio C, 44 – 1989: 1–64.

[B11] Dyar HG (1890) The number of molts of lepidopterous larvae. Psyche 5: 420–422. 10.1155/1890/23871

[B12] Gosik R Łętowski J (2003) Ryjkowcowate (Curculionoidea: Rhinomaceridae, Attelabidae, Apionidae, Curculionidae) użytku ekologicznego „Biała Góra”. Parki Narodowe i Rezerwaty Przyrody 21(2): 247–266.

[B13] Gosik R Łętowski J Kozak E (2010) Morphology of the mature larva and pupa of *Diplapion confluens* (Kirby, 1808) (Coleoptera: Apionidae). Polish Journal of Entomology 79: 211–221.

[B14] Łętowski J (1991) Morfologia i biologia trzech gatunków z rodzaju *Apion* Herbst (Apionidae, Coleoptera) uszkadzających sparcetę siewną (*Onobrychis viciaefolia* Scop.). Wydawnictwo Uniwesytetu Marii Curie-Skłodowkiej, 95 pp.

[B15] Łętowski J (2008) The weevils (Curculionoidea) of calcacerous habitats of the vicinity of Chełm. Teka Komisji Ochrony i Kształtowania Środowiska Przyrodniczego OL PAN 5: 5–125.

[B16] Łętowski J Gosik R Czarniawski W Budzyńska E (2003) Materiały do znajomości ryjkowcowatych (*Curculionoidea*, *Coleoptera*) Kazimierskiego Parku Krajobrazowego. Parki Narodowe i Rezerwaty Przyrody 22(2): 227–245.

[B17] Łętowski J Pawlęga K Ścibior R Rojek K (2015) The morphology of the preimaginal stages of *Squamapion elongatum* (Germar, 1817) (Coleoptera, Curculionoidea, Apionidae) and notes on its biology. ZooKeys 519: 101–115. 10.3897/zookeys.519.9134 PMC4591605 26448708

[B18] Marvaldi AE (1998) Larvae of Entiminae (Coleoptera: Curculionidae): tribal diagnoses and phylogenetic key, with a proposal about natural groups within Entimini. Entomologica Scandinavica 29: 89–98. 10.1163/187631298X00212

[B19] Marvaldi AE (1999) Morfología larval en Curculionidae (Insecta: Coleoptera). Acta Zoológica Lilloana 45(1): 7–24.

[B20] Marvaldi AE (2003) Key to larvae of the South American subfamilies of weevils (Coleoptera: Curculionoidea). Revista Chilena de Historia Natural 76: 603–612. 10.4067/S0716-078X2003000400005

[B21] Marvaldi AE Lanteri AA (2005) Key to higher taxa of South American weevils based on adult characters (Coleoptera, Curculionoidea). Revista Chilena de Historia Natural 78: 65–87. 10.4067/S0716-078X2005000100006

[B22] Mokrzycki T Wanat M (2005) A new checklist of the weevils of Poland (Coleoptera: Curculionoidea). Genus 16(1): 69–117.

[B23] Oberprieler RG Anderson RS Marvaldi AE (2014) Curculionoidea Latreille, 1802: Introduction, Phylogeny. In: Leschen RAB Beutel RG (Eds) Handbook of Zoology (Vol.3), Walter de Gruyter GmbH, Berlin, Boston, 285–300.

[B24] Petryszak B (2004) Curculionoidea. In: Bogdanowicz W, Chudzicka E, Pilipiuk I, Skibińska E (Eds) Fauna of Poland. Characteristics and Checklist of Species. Museum i Instytut Zoologii PAN (Vol. I). Warszawa, 509 pp.

[B25] Scherf H (1964) Die Entwicklungsstadien der mitteleuropäischen Curculioniden (Morphologie, Bionomie, Ökologie). Verlag Waldemar Kramer, Frankfurt am Main, 335 pp.

[B26] Wanat M (1997) New and little known *Squamapion* species (Coleoptera: Apionidae) from western Palaearctic. Annales Zoologici 47(1/2): 285–295.

[B27] Wang Z Alonso-Zarazaga MA Zhou D Zhang R (2013) A description of preimaginal stages of *Pseudaspidapion botanicum* Alonso-Zarazaga & Wang, 2011 (Apionidae, Curculionoidea). ZooKeys 260: 49–59. 10.3897/zookeys.260.4450 PMC3591775 23653504

[B28] Zacharuk RY (1985) Antennae and sensilla. In: Kerkut GA and Gilbert LI (Eds) Comparative Insects Physiology, Pergamon Press, Oxford, Chemistry and Pharmacology 6: 1–69.

